# Mise au point sur le kyste arachnoïdien intrasellaire: à propos d’un cas

**DOI:** 10.11604/pamj.2019.34.55.18564

**Published:** 2019-09-27

**Authors:** Maguette Mbaye, Nfamara Sylla, Mbaye Thioub, Elhadj Cheikh Ndiaye Sy, Mohameth Faye, Alioune Badara Thiam, Momar Code Ba, Seydou Boubakar Badiane

**Affiliations:** 1 Service de Neurochirurgie, Centre Hospitalier National Universitaire de Fann, Université Cheikh Anta Diop, Dakar, Sénégal; 2 Service de Neurochirurgie, Hôpital Général de Grand Yoff, Dakar, Sénégal

**Keywords:** Kyste arachnoïdien, intrasellaire, endoscopie, Arachnoid cyst, intrasellar, endoscopy

## Abstract

Les kystes arachnoïdiens intrasellaires sont des malformations bénignes. La localisation intrasellaire est extrêmement rare de l’ordre de 3%. Leur physiopathologie est encore mal élucidée. Nous rapportons un cas de kyste arachnoïdien intrasellaire à expansion suprasellaire dont la prise en charge avait consisté en une fenestration endoscopique par voie transsphénoïdale. Les aspects épidémiologiques, cliniques, physiopathologiques, radiologiques, thérapeutiques et évolutifs ont été analysés. Les procédures neuroendoscopiques sont de plus en plus utilisées pour la prise en charge chirurgicale. Leur pronostic est bon, et la récidive fréquente même après plusieurs années d’évolution.

## Abstract (en)

Intrasellar arachnoid cysts are benign malformations. They are extremely rare (approximately 3% of cases). Their pathophysiology is still poorly elucidated. We here report a case of intrasellar arachnoid cyst with suprasellar extension whose treatment was based on endoscopic transsphenoidal fenestration. The epidemiological, clinical, pathophysiological, radiological, therapeutic and evolutionary features have been analyzed. Neuroendoscopic procedures are performed with increasing frequency in surgery. Prognosis is good and recurrences are frequent, even after several years of evolution.

## Introduction

Les kystes arachnoïdiens sont des malformations bénignes de l’arachnoïde. Ils peuvent se développer partout où il existe de l’arachnoïde [1]. La localisation intrasellaire est très rare [2], environ 3% de l’ensemble des kystes arachnoïdiens intracrâniens [3]. Les troubles visuels, les céphalées et parfois les troubles hormonaux sont les signes typiques de cette localisation. Cependant, la découverte fortuite n’est pas rare. Le diagnostic radiologique est posé essentiellement par l’imagerie par résonance magnétique (IRM) [4]. Le traitement chirurgical traditionnel des kystes arachnoïdiens intrasellaires comprend différentes options comme la craniotomie avec exérèse du kyste, la dérivation du kyste, l’exérèse associée à la dérivation, la fenestration endoscopique et la ventriculo-kystostomie percutanée [5]. L’introduction de la neuroendoscopie a procuré une modalité moins invasive du traitement chirurgical de ces kystes. Nous rapportons une observation clinique d’un kyste arachnoïdien intrasellaire à expansion suprasellaire et une revue de la littérature.

## Patient et observation

**Figure 1:**
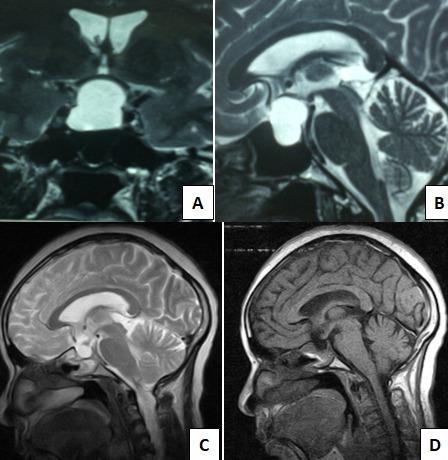
IRM préopératoire coupes coronale T2 (A) et sagittale T2 (B) montrant un kyste arachnoïdien intrasellaire à extension suprasellaire refoulant le chiasma optique vers le haut; IRM de contrôle confirmant la vidange complète du kyste: (C) (T2) et D (T1)

Il s’agissait d’une patiente de 40 ans, mariée, qui avait consulté, pour des céphalées frontales d’irradiation occipitales intenses et invalidantes, un flou visuel bilatéral et une irrégularité menstruelle à type d’aménorrhée intermittente. La patiente ne présentait pas d’antécédents particuliers. Le bilan hormonal: comportant un dosage des hormones suivantes ACTH, STH, FSH, LH, TSH et PRL qui révélait une élévation des taux de LH et de FSH respectivement à 15,11mUI/l (2-9) et 28,40UI/l (2-12). Le bilan ophtalmologique: composé de fond d’œil, de champ visuel et d’acuité visuel, n’avait pas révélé d’anomalie. L’imagerie par résonnance magnétique (IRM) cérébrale (Figure 1 A, Figure 1 B) avait mis en évidence une lésion kystique intrasellaire à expansion suprasellaire refoulant le chiasma optique et l’hypophyse vers le haut, en hyposignal T1 et en hypersignal T2. Le traitement a consisté en une fenestration endoscopique du kyste par voie transsphénoïdale endonasale. Les suites opératoires ont été marquées par un diabète insipide transitoire contrôlé par l’administration de la desmopressine. A J4 post-opératoire, la patiente était sortie de l’hôpital. L’évolution après un recul de 12 mois, nous avons noté une amélioration de l’état clinique de la patiente par rémission des céphalées et du flou visuel, et la normalisation du cycle menstruel. Le bilan hormonal post-opératoire était normal. L’IRM de contrôle (Figure 1 C, Figure 1 D), effectuée à deux mois de la chirurgie, a confirmé la vidange complète du kyste.

## Discussion

### Définition et physiopathologie

Décrits pour la première fois en 1831 par BRIGHT [6]. Les kystes arachnoïdiens sont des malformations liées à une anomalie de développement dans laquelle le fractionnement ou la duplication de l’arachnoïde primitive conduit à une collection intra-arachnoïdienne du liquide céphalorachidien [7]. La présence d’une grande ouverture du diaphragme sellaire ou encore l’absence de diaphragme, et l’écart entre le volume de la glande hypophysaire et de la selle turcique sont des facteurs qui favorisent la pénétration de l’arachnoïde dans la selle turcique [4]. Il a été démontré que l’espace sous-arachnoïdien peut pénétrer dans la selle turcique dans les conditions normales [3]. Dans les conditions pathologiques, telles que l’hypertension intracrânienne ou l’atrophie de la glande hypophysaire, l’espace sous arachnoïdien peut également pénétrer dans la selle turcique [8, 9].Une grande ouverture du diaphragme combinée à une force pulsatile de liquide céphalo-rachidien (LCR) permet à l’espace sous arachnoïdien de pénétrer dans la selle turcique. La tige pituitaire et l’hypophyse participent alors par un mécanisme de vanne à boisseau sphérique refermant le défect dural après l’entrée de LCR (kyste communiquant). L’apposition des membranes arachnoïdiennes à un moment donné pourrait engendrer un kyste non communiquant. Le mécanisme par lequel les kystes s’étendent n’est pas connu [10]. Bien que certains auteurs estiment qu’une action de valve existe seulement dans les kystes traumatiques [8]. Nous n’avions pas retrouvé de contexte traumatique ancien ou récent chez notre patiente.

### Incidence/fréquence

Les kystes arachnoïdiens sont des malformations retrouvées chez 0,2 à 1,7% de la population, y compris les cas asymptomatiques [2, 11, 12]. En outre, les kystes arachnoïdiens symptomatiques sont rares en particulier chez l’adulte. Ils sont principalement repartis dans la fosse cérébrale moyenne et l’angle ponto-cérébelleux, et sont rarement diagnostiqués en intrasellaire où ils représentent 9-10% de l’ensemble des kystes arachnoïdiens intracrâniens [3]. Sur une période de 56 mois nous avons enregistré deux cas de kyste arachnoïdien intrasellaire sur 65 lésions sellaires, soit 3%. La vulgarisation des explorations neuroradiologiques expliquerait cette augmentation de la fréquence avec une découverte élevée d’incidentalomes.

### Aspects cliniques

La revue de la littérature [1, 6, 13] retrouve les céphalées et les troubles visuels comme signes révélateurs dominants associées à un kyste arachnoïdien intrasellaire. Les signes endocriniens sont moins fréquents; la plupart des plaintes portent sur l’axe gonadotrope, notamment les irrégularités menstruelles, l’infertilité, la baisse de la libido. C’est le cas de notre patiente qui a consulté pour des céphalées, troubles visuels à type de flou visuel et une irrégularité menstruelle à type d’aménorrhée intermittente. Le kyste arachnoïdien intrasellaire se manifeste comme processus expansif à l’intérieur et au-dessus de la fosse hypophysaire entrainant une compression opto-chiasmatique, une diminution de la fonction de l’hypophyse antérieure ou des céphalées. Ces céphalées peuvent découler de la distension de la dure-mère causée par le kyste. Dans certains cas, ils ne sont probablement pas liés au kyste, ce dernier étant découvert de façon fortuite, au cours des explorations pour d’autres pathologies [14].

### Diagnostic radiologique

Le diagnostic d’un kyste arachnoïdien intrasellaire préopératoire est difficile. Le diagnostic différentiel se fait avec de multiples lésions de la région sellaire et suprasellaire tels que les adénomes hypophysaires nécrosés, les craniopharyngiomes kystiques, le kyste de la poche de Rathke etc. L’IRM est l’examen de choix qui permet de l’évoquer de façon formelle, en précisant la densité du contenu du kyste et la présence ou non d’une prise de contraste périphérique [11, 14]. Le diagnostic de kyste arachnoïdien intrasellaire doit être évoqué devant toute formation de nature kystique dont les densités et le signal apparaissent, en tout point, identiques à ceux du liquide céphalorachidien, même s’il existe des prises de contraste au niveau de la paroi du kyste. Ces prises de contraste peuvent être liées à la compression de la tige pituitaire et au déplacement de l’hypophyse normale qui peut se rehausser [15]. Chez notre patiente, le diagnostic a été évoqué devant une lésion intrasellaire à extension suprasellaire refoulant le chiasma optique et l’hypophyse vers le haut, sans rehaussement périphérique. Hypo intense sur la séquence T1 et hyper intense sur les séquences T2.

### Traitement

Les modalités de la gestion chirurgicale des kystes arachnoïdiens intrasellaires et les résultats sont principalement influencés par la relation entre le kyste lui-même et les citernes de la base et par la présence d’une hydrocéphalie associée [16]. Le traitement chirurgical est indiqué chez les patients symptomatiques ou lorsque le kyste évolue. Diverses procédures chirurgicales et voies d’abord ont été proposées comprenant l’abord direct par craniotomie, la dérivation du kyste, la fenestration endoscopique. Le manque de critères préopératoires fiables qui permettent de prédire les résultats de ces procédures peut expliquer pourquoi la plupart des chirurgiens ont souvent traité leurs cas personnels avec différentes techniques chirurgicales. Cependant, il n’y a pas de rapports définissant le meilleur traitement chirurgical en termes de résultats [17].

### Abord direct

L’abord direct du kyste, selon certains auteurs [16], a été effectué par les voies sous frontale, pterionale, transventriculaire ou transcalleuse afin de réaliser une excision de la paroi du kyste, une fenestration ou une marsupialisation. Ce traitement est relativement invasif, en raison de la profondeur du kyste. RAFFAEL et MCCOMB [17] ont rapporté que 75% de leurs patients, sans hydrocéphalie associée, ont été traités avec succès par cette procédure seule. Cependant, la récidive a été fréquente, en particulier dans les cas traités par la voie sous frontale et fenestration ou marsupialisation. Selon certains [5, 12], la suspicion de kyste arachnoïdien intrasellaire à expansion suprasellaire modifiera systématiquement l’approche chirurgicale qui devra plutôt se faire par voie sous frontale. Certains auteurs [18, 19] avaient souligné les bons résultats obtenus par une approche transcalleuse, car le dôme du kyste a été ouvert dans le ventricule latéral. Dans l’étude de Gangemi *et al.* [16], 79% des patients ont été guéris après un abord direct seul. Cependant, un quart des patients avaient, plus tard, nécessité une dérivation du kyste ou de l’hydrocéphalie associée ou les deux. Les causes de l’échec seraient une ouverture et une résection insuffisantes de la paroi du kyste, l’incapacité de la citerne chiasmatique à s’adapter au détournement de liquide céphalorachidien du kyste, le manque de communication entre le kyste et le système ventriculaire ou une anomalie généralisée de l’absorption du liquide céphalorachidien [4, 10, 17].

### Dérivation du kyste

La dérivation du kyste est une technique moins invasive et plus sûre par rapport à l’approche microchirurgicale directe [18, 19]. Le taux de réussite selon GANGEMI [16] est de 85,7%. La principale cause de l’échec est que la paroi du kyste ne peut pas s’effondrer, car elle est souvent plus épaisse et plus rigide que celle des kystes arachnoïdiens d’autres localisations [13]. Ainsi, certains patients ont par la suite besoin d’une dérivation ventriculaire pour traiter l’hydrocéphalie. L’inconvénient de la dérivation du kyste est le dysfonctionnement ou l’infection.

### Fenestration endoscopique transventriculaire

L’endoscope permet la fenestration du kyste à la fois dans le système ventriculaire et les citernes de la base sous contrôle visuel et d’une manière plus sûre et plus facile qu’avec la ventriculo kystostomie percutanée [16, 20]. Les kystes arachnoïdiens supra sellaire qui subissent un traitement chirurgical sont toujours grands et associés à une hydrocéphalie; ainsi, la paroi du kyste faisant saillie à travers le foramen de Monro peut être facilement accessible et fenestrée. Il en résulte une détente du kyste et recanalisation partielle du troisième ventricule. Une seconde fenestration doit également être effectuée entre le kyste et la citerne pré pontique pour assurer l’écoulement de liquide céphalorachidien dans les espaces sous arachnoïdiens [16, 20]. Cette procédure a entrainé une guérison ou une amélioration dans 85,7% des cas. Lorsqu’elle est effectuée, réalisant ainsi une ventriculo kysto cisternostomie, elle restaure la circulation du LCR entre le kyste et les citernes de la base. Ce qui diminue le risque de récidive. Dans la série de GANGEMIE [16], 94,3% des patients ont été guéris ou améliorés par cette technique. La neuronavigation peut être appoint utile dans la fenestration transventriculaire d’un kyste arachnoïdien suprasellaire surtout quand il n’y a pas de dilatation ventriculaire marquée.

### Fenestration transsphénoïdale

Les indications du traitement par cette voie sont les mêmes que celui d’un adénome non secrétant à savoir: hypertension intracrânienne, troubles visuels et troubles endocriniens [10, 18]. Les objectifs sont d’évacuer le liquide céphalorachidien du kyste et d’exciser la totalité ou en partie les parois du kyste et si possible créer une communication avec les espaces sous arachnoïdiens suprasellaires. L’excision de la paroi du kyste permet un diagnostic histologique et ne doit pas être traumatisante pour l’hypophyse. En complément de la procédure, le plancher de la selle turcique doit être reconstruit hermétiquement et les sinus sphénoïdaux doivent être rembourrés efficacement avec de la graisse pour prévenir la fuite de liquide céphalorachidien; ou alors fermer la dure-mère avec des points séparés à l’aide du nylon 6/0 comme recommandent certains [21]. Nous n’avons pas noté de fuite de liquide céphalorachidien dans notre observation. Les rhinorrhées cérébrospinales sont signalées dans la littérature [4, 13, 17, 19] et la plupart d’entre elles ont nécessité une ré-intervention. Une autre étude [2] rapporte que huit de ses 31 patients ont présenté des complications à type de rhinorrhée, d’infection et de cécité, et deux étaient décédés de méningite. Cette grave morbidité illustre l’importance d’une fermeture méticuleuse de la selle turcique et des sinus sphénoïdaux, associée à une hypotension intracrânienne posturale en post- opératoire et une ponction lombaire pendant plusieurs jours [4]. Cette cécité post-opératoire pourrait être expliquée par un prolapsus du chiasma optique dans une selle turcique vidée. Elle peut être prévenue par un bourrage efficace lors de la fermeture par de la graisse [4, 18]. Chez notre patiente nous avions une nette amélioration des troubles visuels en post-opératoire immédiat. Il est par ailleurs recommandé dans la littérature d’utiliser la voie transsphénoïdale, moins invasive, pour des petits kystes arachnoïdiens intrasellaires avec packing étanche de la fosse hypophysaire sans faire communiquer avec les espaces sous arachnoïdiens suprasellaires, et une craniotomie pour des kystes arachnoïdiens intrasellaires larges pour éviter des complications et créer une communication entre le kyste et espaces sous arachnoïdiens [14, 21]. L’évaluation post-opératoire des résultats après les procédures endoscopiques peut être réalisée à la fois par les paramètres cliniques et radiologiques. La rémission des signes d’hypertension intracrânienne et l’amélioration des troubles visuels sont observés dans la majorité des cas, alors que les perturbations du système endocrinien ne sont pas souvent parfois améliorées [16, 20, 21], contrairement à notre étude où nous notons une amélioration des troubles menstruels par la reprise normale du cycle menstruel. Les caractéristiques de l’IRM associées à un bon résultat sont la diminution du déplacement du chiasma et de l’effacement pontique et le remplissage du troisième ventricule. Il est à signaler que la récidive est possible par cette voie, même après plusieurs années [6].

## Conclusion

Les kystes arachnoïdiens intrasellaires sont des malformations bénignes. La localisation intrasellaire est extrêmement rare dans l’ordre de 3%. Leur physiopathologie est mal élucidée. Les céphalées et les troubles visuels sont des symptômes révélateurs associés aux kystes intrasellaires et dans un certain nombre réduit des cas, des troubles hormonaux. L’exploration des kystes arachnoïdiens intrasellaires par l’IRM permet d’apprécier ses rapports avec les structures optiques et la glande hypophysaire. Le traitement chirurgical, de la craniotomie à la fenestration endoscopique, suscite encore des controverses. Cependant, les procédures neuroendoscopiques sont de plus en plus utilisées. Moins de cent cas ont été décrits dans la littérature internationale néanmoins le pronostic des kystes arachnoïdiens intrasellaires reste bon. Un suivi à long terme après la chirurgie est alors nécessaire étant donné le risque de récidive même après plusieurs années.

## Conflits d’intérêts

Les auteurs ne déclarent aucun conflit d'intérêts.

## References

[1] Nomura M, Tachibana O, Hasegawa M, Kohda Y, Nakada M, Yamashima T, Yamashita J, Suzuki M (1996). contrast-enhanced MRI of intrasellar arachnoid cysts: relationship between the pituitary gland ant cyst. Neuroradiology.

[2] Miyamoto T, Ebisudani D, Kitamura K, Ohshima T, Horiguchi H, Nagahiro S (1999). Surgical management of symptomatic intrasellar arachnoid cysts--two case reports. Neurol Med Chir (Tokyo).

[3] Rengachary SS, Watanabe I (1981). Ultrastructure and pathogenesis of intracranial arachnoid cysts. J Neuropathol Exp Neurol.

[4] Dubuisson AS, Stevenaert A, Martin DH, Flandroy PP (2007). Intrasellar arachnoid cysts. Neurosurgery.

[5] Pierre-Kahn A, Capelle L, Brauner R, Sainte-Rose C, Renier D, Rappaport R, Hirsch JF (1990). Presentation and management of suprasellar arachnoid cysts.S Review of 20 cases. J Neurosurg.

[6] Murakami M, Okumura H, Kakita K (2003). Recurrent intrasellar arachnoid cyst. Neurol Med Chir (Tokyo).

[7] Wang X, Chen JX, You C, Jiang S (2012). CT cisternography in intracranial symptomatic arachnoid cysts: Classification and treatment. J Neurol Sci.

[8] Shim KW, Park EK, Lee YH, Kim SH, Kim DS (2013). Transventricular Endoscopic Fenestration of Intrasellar Arachnoid Cyst. Neurosurgery.

[9] Adeeb N, Deep A, Griessenauer CJ, Mortazavi MM, Watanabe K, Loukas M, Tubbs RS, Cohen-Gadol AA (2013). The intracranial arachnoid mater: a comprehensive review of its history, anatomy, imaging, and pathology. Childs Nerv Syst.

[10] Schroeder HW, Gaab MR (1997). Endoscopic observation of a slit-valve mechanism in a suprasellar prepontine arachnoid cyst: case report. Neurosurgery.

[11] Eskandary H, Sabba M, Khajehpour F, Eskandari M (2005). Incidental findings in brain computed tomography scans of 3000 head trauma patients. Surg Neurol.

[12] Katzman GL, Dagher AP, Patronas NJ (1999). Incidental findings on brain magnetic resonance imaging from 1000 asymptomatic volunteers. JAMA.

[13] Tamburrini G, D'Angelo L, Paternoster G, Massimi L, Caldarelli M, Di Rocco C (2007). Endoscopic management of intra and paraventricular CSF cysts. Childs Nerv Syst.

[14] Weber F, Knopf H (2006). Incidental findings in magnetic resonance imaging of the brains of healthy young men. J Neurol Sci.

[15] Dietemann JL, Guessoum M, Schultz A, Zöllner G, Sanoussi S, Maitrot D, Buchheit F (1997). Kystes arachnoïdiens intrasellaires: scanographie et IRM, à propos de deux observations. J Neuroradiologie.

[16] Gangemi M, Colella G, Magro F, Maiuri F (2007). Suprasellar arachnoid cysts: endoscopy versus microsurgical cyst excision and shunting. Br J Neurosurg.

[17] Raffael C, McComb JG (1988). To shunt or to fenestrate: Which is the best surgical treatment for arachnoid cysts in paediatric patients?. Neurosurgery.

[18] Güdük M, HamitAytar M, Sav A, Berkman Z (2016). Intrasellar arachnoid cyst: a case report and review of the literature. Int J Surg Case Rep.

[19] Hoffman HJ, Hendrick EB, Humphreys RP, Armstrong EA (1982). Investigation and management of suprasellar arachnoid cysts. J Neurosurg.

[20] André A, Zérah M, Roujeau T, Brunelle F, Blauwblomme T, Puget S (2016). Suprasellar Arachnoid Cysts: Toward a New Simple Classification Based on Prognosis and Treatment Modality. Neurosurgery.

[21] Su Y, Ishii Y, Lin CM, Tahara S, Teramoto A, Morita A (2015). Endoscopic Transsphenoidal Cisternostomy for Nonneoplastic Sellar Cysts. Biomed Res Int.

